# Extract from *Aphloia theiformis*, an edible indigenous plant from Reunion Island, impairs Zika virus attachment to the host cell surface

**DOI:** 10.1038/s41598-018-29183-2

**Published:** 2018-07-18

**Authors:** Elodie Clain, Laura Sinigaglia, Andrea Cristine Koishi, Olivier Gorgette, Gilles Gadea, Wildriss Viranaicken, Pascale Krejbich-Trotot, Patrick Mavingui, Philippe Desprès, Claudia Nunes Duarte dos Santos, Pascale Guiraud, Nolwenn Jouvenet, Chaker El Kalamouni

**Affiliations:** 1 Université de La Réunion, UM134 Processus Infectieux Insulaire Tropical (PIMIT), INSERM U1187, CNRS UMR 9192, IRD UMR 249, Plateforme Technologique CYROI, 97490 Sainte, Clotilde France; 2 UMR CNRS 3569, Viral Genomics and Vaccination Unit, Pasteur Institute, 75724 Paris, France; 3 Laboratorio de Virologia Molecular, Instituto Carlos Chagas, ICC/FIOCRUZ/PR, Curitiba, Parana Brazil; 4 Ultrastructural BioImaging (UTechsUBI), Pasteur Institute, 75724 Paris, France

## Abstract

The mosquito-borne Zika virus (ZIKV) belongs to the flavivirus genus of the *Flaviviridae* family. Contemporary epidemic strains of ZIKV are associated with congenital malformations in infants, including microcephaly, as well as Guillain-Barré syndrome in adults. A risk of human-to-human transmission of ZIKV is also well documented. A worldwide research effort has been undertaken to identify safe and effective strategies to prevent or treat ZIKV infection. We show here that extract from *Aphloia theiformis*, an edible endemic plant from Indian Ocean islands, exerts a potent antiviral effect against ZIKV strains of African and Asian lineages, including epidemic strains. The antiviral effect of *A*. *theiformis* extract was extended to clinical isolates of dengue virus (DENV) of the four serotypes in human hepatocytes. *A*. *theiformis* inhibited virus entry in host cells by acting directly on viral particles, thus impairing their attachment to the cell surface. Electron microscopic observations revealed that organization of ZIKV particles was severely affected by *A*. *theiformis*. We propose a model of antiviral action for *A*. *theiformis* against flaviviruses that highlights the potential of medicinal plants as promising sources of naturally-derived antiviral compounds to prevent ZIKV and DENV infections.

## Introduction

Zoonotic Zika virus (ZIKV) is a mosquito-borne virus that emerged in 2007 in Micronesia and since has caused important outbreaks in the South Pacific, Americas and South-East Asia. These recent ZIKV epidemics were associated with severe fetal brain injuries and neurological defects in adults, such as Guillain-Barre syndrome^1,2^. ZIKV infection is now identified as a sexually-transmitted illness as well^3–5^. In 2016, Zika infection was declared an emerging epidemic threat worldwide by the World Health Organization.

ZIKV is a member of the flavivirus genus, a group of small, enveloped viruses, which also includes Dengue virus (DENV), West Nile virus (WNV) and Yellow fever virus^6^. The genome consists of a single-stranded, positive-sense RNA molecule of around 10,7 kb, encoding a polyprotein precursor that is processed by the viral protease NS3 to give rise to 7 non-structural (NS) proteins and 3 structural proteins (Capsid C, pre-membrane prM and Envelope E). The NS proteins are mainly involved in viral RNA replication, while the structural proteins constitute the virion^7,8^.

The early stages of ZIKV infection require the attachment of the virion to the cell surface. This first step is mainly mediated by the interaction between phosphatidylserine exposed at the surface of the virus and the cellular receptor Axl^9^ and probably also mobilizes close contacts between the E protein and the cell membrane. Following Axl mediated-binding, the virus enters target cells through clathrin-mediated endocytosis^9^. The low-pH environment of endosomes triggers fusion between the viral envelope and the endosomal membrane. This fusion event leads to the release of the viral nucleocapsid into the cytosol.

To date, there is still no vaccination or specific treatment available for ZIKV. Therefore, it is of utmost urgency to develop safe and effective anti-ZIKV compounds, not only to mitigate ZIKV-associated morbidities but also to impair the chain of transmission. The features of E mediated events make the development of entry inhibitors an attractive possibility^10^. Medicinal plants, which have been used as treatment or prevention against human diseases for millenaries, remain a remarkable source of potential antiviral compounds. Indeed, numerous enveloped RNA viruses are sensitive to a broad range of phytochemicals, including alkaloids, coumarins, flavonoids, terpenoids, polyphenols and saponins^11,12^. It has been recently reported that ZIKV is sensitive to polyphenol epigallocatechin gallate (EGCG) from green tea and to curcumin^13–15^. The Reunion Island which belongs to the Mascarene Archipelago, is described as a biodiversity hotspot, based on its remarkable flora and endemic species^16^. Previous studies have shown that some edible and medicinal plants from Reunion island exert remarkable antioxidant activities due to their high-content of polyphenols, alkaloids and saponins, such as *Aphloia theiformis* (*A*. *theiformis*), *Hubertia ambavilla* (*H*. *ambavilla*) and *Ayapana triplinervis* (*A*. *triplinervis*)^17–20^. Hence, we evaluated whether solvent-free extracts of *A*. *theiformis*, *H*. *ambavilla and A*. *triplinervis*, which were recently listed into the French *pharmacopoeia*, prevents ZIKV infection *in vitro*.

## Results

### *A*. *theiformis* extract inhibits the early stage of ZIKV infection

**Figure 1:**
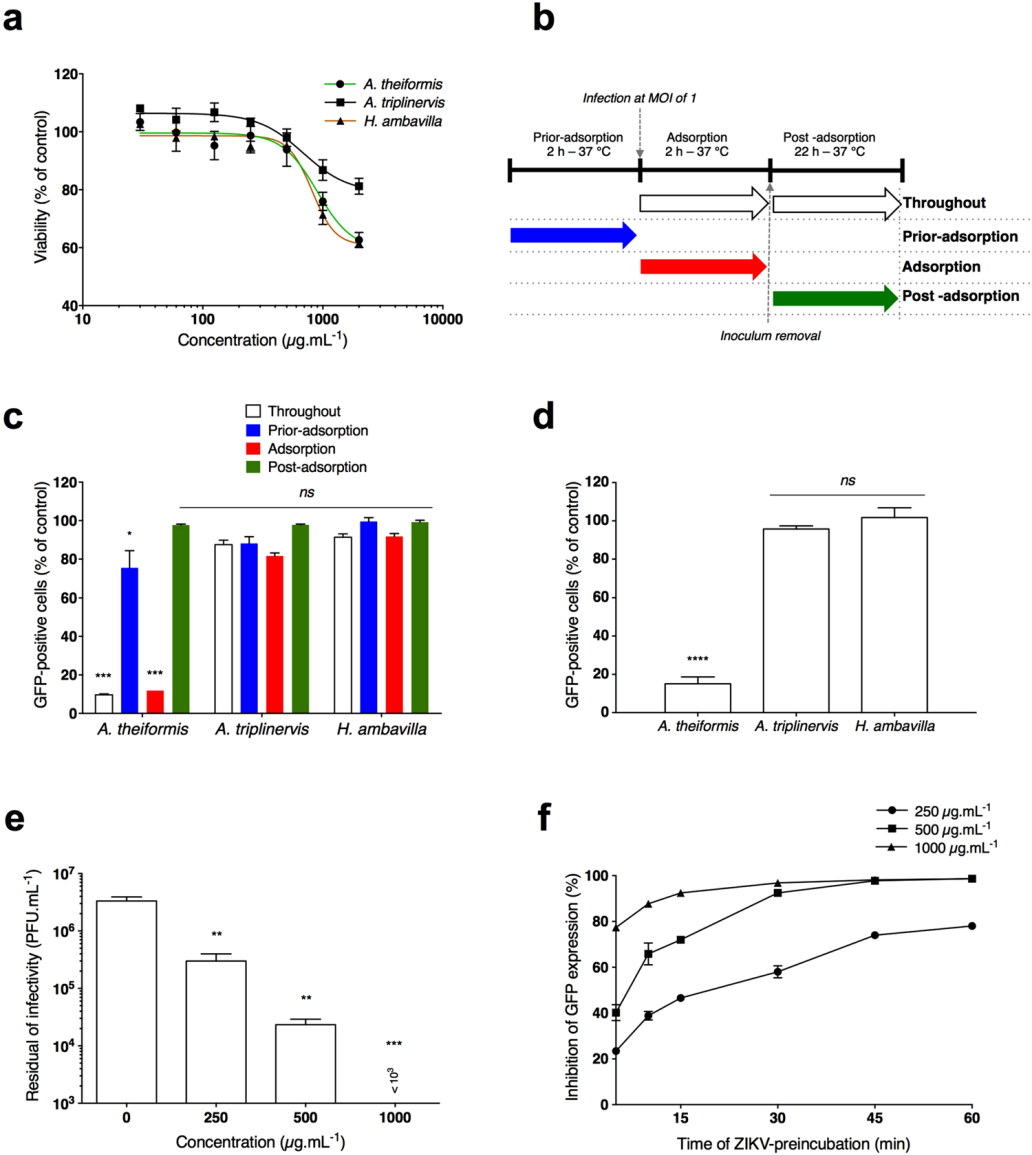
*A*. *theiformis* extract targets early stages of ZIKV replication cycle. (**a**) Viability of Vero cells incubated with different concentrations of plant extracts. Cells were cultured in the presence of increased concentrations of plant extracts for 72 h. Cell metabolic activity was evaluated by MTT assay. Results are means ± SD of four independent experiments and are expressed as relative value compared to untreated cells. (**b**) Schematic representation of time-of-drug addition assay used to characterise antiviral activity of the plant extracts (500 µg.mL^−1^) on ZIKV_GFP_ infection of Vero cells. Arrows indicate the presence of plant extract during the infection. (**c**) Flow cytometric analysis of GFP expression in Vero cells infected with ZIKV_GFP_ at MOI of 1 under the experimental conditions shown in (b). Results are means ± SD of four independent experiments and are expressed as relative value compared to untreated infected cells. (**d**) Vero cells were infected with ZIKV_GFP_ pre-incubated during 1 h at 37 °C with plant extracts (500 µg.mL^−1^). Flow cytometric analysis of GFP fluorescence was performed 24 hpi. The results shown are means ± SD of four independent experiments are expressed as relative value compared to untreated infected cells. (**e**) ZIKV_GFP_ was incubated with three different concentrations of *A*. *theiformis* and the residual infectious particles were titrated by plaque forming assay. The results shown are means ± SD of four independent experiments. (**f**) GFP expression in Vero cells infected with ZIKV_GFP_ (MOI 1) pre-incubated for various times with three different concentrations of *A*. *theiformis* at 37 °C. Flow cytometric analysis of GFP fluorescence was performed 24 hpi. The relative percentages of inhibition are means ± SD of four independent experiments. One-way ANOVA and Dunnett’s test for multiple comparisons (*p < 0.05; **p < 0.01; ***p < 0.001, ****p < 0.0001, ns = not significant compared to untreated control).

Prior to evaluate the anti-ZIKV properties of extracts from *A*. *theiformis*, *A*. *triplinervis* and *H*. *ambavilla*, we determined their maximal non-cytotoxic doses on Vero cells using a MTT assay, which assesses cell metabolic activity (Fig. 1a). Plotting cell viability against different concentrations of plant extracts revealed concentration-dependent toxicity in Vero cells (Fig. 1a). The concentrations that inhibited 50% of cell viability (CC_50_) were up to 3000 µg.mL^−1^ for all tested plant extracts. A concentration of 500 µg.mL^−1^ of plant extracts that maintained 95% of cell viability (Fig. 1a) was chosen for testing potential anti-ZIKV activity.

Time-of-drug addition approach was performed to determine which stages of ZIKV infection could be targeted by the 3 medicinal plants extracts (Fig. 1b). A chimeric molecular clone of the ZIKV strain MR766 expressing a GFP reporter gene as an additional part of the structural protein region (ZIKV_GFP_)^21^ was used for monitoring viral replication by flow cytometry in Vero cells. The number of GFP positive cells decreased around 90% compared to non-treated control cells when 500 µg.mL^−1^ of *A*. *theiformis* extract was added throughout the experiment (Fig. 1b and c). Similar results were obtained when *A*. *theiformis* extract was added concomitantly with virus input (adsorption) for 2 hours (Fig. 1b and c). In contrast, little or no antiviral effect was observed when *A*. *theiformis* extract was added *prior* adsorption or after virus exposure (Fig. 1b and c). Treatment of cells with *H*. *ambavilla* and *A*. *triplinervis* showed no significant effect on ZIKV_GFP_ replication regardless of the experimental conditions tested (Fig. 1c). These results suggest that *A*. *theiformis*-mediated inhibition of ZIKV infection was not associated with an impairment of viral replication but rather to an inability of ZIKV to initiate a productive infection into the host-cell.

To investigate whether *A*. *theiformis* extract renders virus particles unable to initiate viral infection in the host-cell, viral inactivation assay was performed. ZIKV_GFP_ preparation was incubated with 500 µg.mL^−1^ of *A*. *theiformis* extract for 1 hour at 37 °C prior to Vero cells infection. Such treatment of ZIKV_GFP_ resulted in around 85% reduction of GFP-positive cells at 24 hours post-infection (hpi) compared to cells infected plant-extract-free ZIKV_GFP_ (Fig. 1d). The infectivity of ZIKV_GFP_ treated with *H*. *ambavilla* and *A*. *triplinervis* extracts was similar to that of non-treated virus (Fig. 1d), further suggesting that these 2 plants do not possess anti-ZIKV activities.

We next wondered whether the antiviral activity of *A*. *theiformis* extract was affect by the extraction process (Fig. S1a). Three extractions were performed using plants harvested at different times in the year 2016 and their antiviral activity was evaluated. Our data showed that the antiviral activity of *A*. *theiformis* was not affected by the extraction itself, suggesting that the concentration of the active components is stable and does not vary from one extraction to the other.

To ensure that *A*. *theiformis* extract did not exert non-lethal cytotoxic effects that would have been missed in the MTT assays (Fig. 1a), the effect of 500 µg.mL^−1^ of *A*. *theiformis* extract on the growth of Vero cells was evaluated using flow cytometry analysis (Fig. S1b). Our data showed that *A*. *theiformis* extract did not affect the growth of Vero cells. Moreover, to evaluate the potential cytotoxic effect of *A*. *theiformis* extract on human cells, MTT cell viability assays were performed on the human hepatoma cell lines Huh7.5, which are extensively used in *Flaviviridae* research (Fig. S1c). A concentration of 500 µg.mL^−1^ of plant extract maintained 100% of Huh7.5 cells viability (Fig. S1c). We conclude that the antiviral activity of the compound is not due to its cytotoxicity.

To establish a causal relationship between the reduced percentage of GFP-positive cells in the samples treated with *A*. *theiformis* extract and a loss of ZIKV infectivity, the presence of infectious viral particles in the inoculum was titrated by plaque-forming assay (Fig. 1e). A dose-dependent effect of *A*. *theiformis* on ZIKV infectivity was observed, with a 2-log reduction in infectivity of viral inoculum at non-cytotoxic concentrations of plant extract (Fig. 1e). A complete inhibition of ZIKV infectivity was observed in the presence of 1000 µg.mL^−1^ of *A*. *theiformis* extract (Fig. 1e). The concentration of *A*. *theiformis* extract that inhibited 50% of viral infectivity (IC_50_) was 100 µg.mL^−1^, obtained using nonlinear regression following the construction of a sigmoidal concentration-response curve (Fig. 1e). Based on the determined cytotoxicity and antiviral efficacy (SI = CC_50_/IC_50_), the Selectivity Index of *A*. *theiformis* extract is 30.

To determine the kinetic of *A*. *theiformis* extract-mediated inactivation of ZIKV, a time-response curve was performed on Vero cells. Only ten to fifteen minutes were required to inhibit GFP expression by 50% at non-cytotoxic concentrations of *A*. *theiformis* extract (Fig. 1f). Together, these results suggest that *A*. *theiformis* acts by interfering with at least one of the early steps of ZIKV infection, such as attachment to cell membrane, receptor-mediated cell entry, fusion and/or decapsidation within the cell cytoplasm.

### *A*. *theiformis* inhibits both historical African and contemporary Asian ZIKV strains

**Figure 2:**
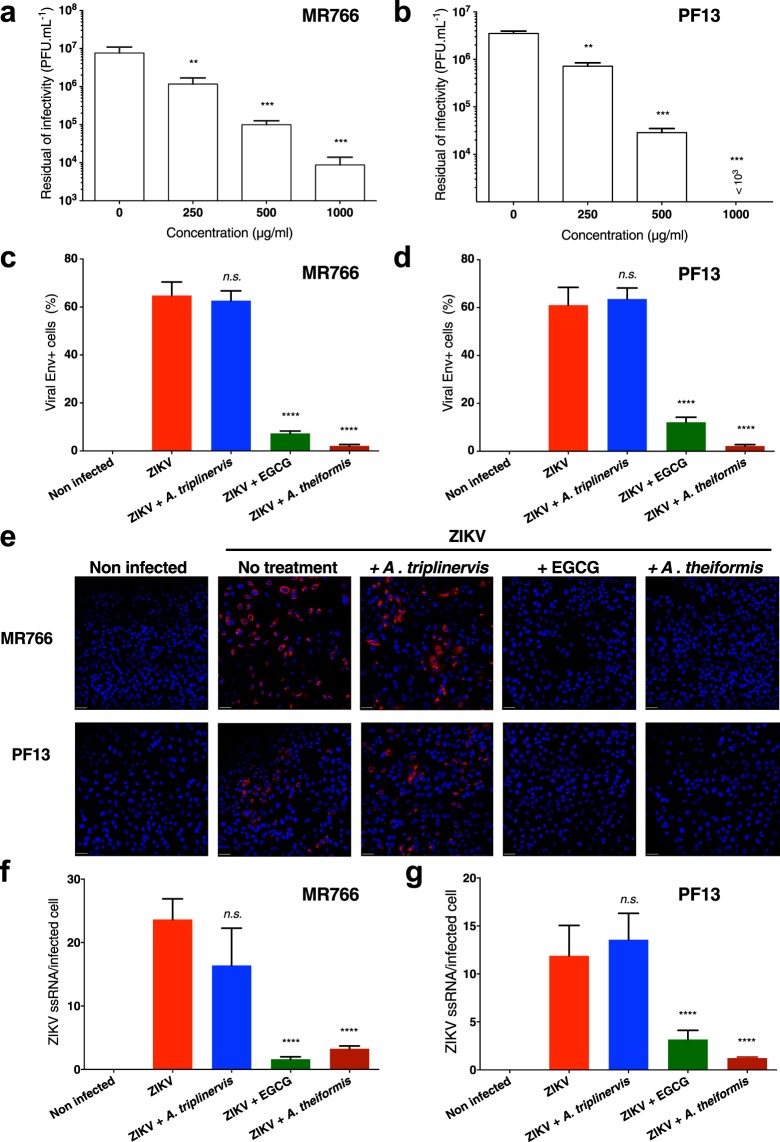
*A*. *theiformis* extract exerts a potent antiviral effect against an African and an Asian ZIKV strains. ZIKV-MR766 or ZIKV-PF13 were incubated with *A*. *theiformis* extract for 1 h at 37 °C. Vero cells were left uninfected or were infected at an MOI of 1 for 24 h. (**a**,**b**) The infectious released particles were titrated by plaque forming assay. The results shown are means ± SD of four independent experiments. (**c**,**d**) Quantification of the number of cells positive for the viral protein E expression in ZIKV-infected Vero cells by immunofluorescence and confocal microscopy. *A*. *triplinervis* and EGCG (100 µM) were used as negative and positive controls, respectively. The results shown are means ± SD of four independent experiments. (**e**) Vero cells were processed for FISH using a probe specific for viral RNA (red) and then stained with NucBlue to visualize nuclei (blue). Images are representative of three independent experiments. Scale bars are 50 m. (**f**,**g**) Quantification of the percentage of Vero cells positive for ssRNA from the experiments represented in e. Data are means ± SD of three independent experiments.

To further validate the role of *A*. *theiformis* extract on ZIKV replication, the African lineage virus strain ZIKV-MR766^22^ and the contemporary Asian lineage strain ZIKV-PF13^23^, which was responsible for the 2015 epidemic in French Polynesia, were incubated with *A*. *theiformis* extract for 1 hour at 37 °C prior to Vero cells infection. Quantification of viral infectivity by plaque-forming assay revealed that both viral strains were as sensitive as ZIKV_GFP_ to different *A*. *theiformis* extract concentrations (Fig. 2a,b). Indeed, as observed for ZIKV_GFP_ (Fig. 1e), *A*. *theiformis* extract lowered infectivity of the two viral strains up to 2-log at non-cytotoxic dose (Fig. 2a,b). Immunofluorescence assays using antibodies that recognize the viral protein E were performed on Vero cells infected for 24 hours with the two viral strains. Epigallocatechin gallate (EGCG)^13^ and *A*. *triplinervis* were used as positive and negative controls, respectively. The analysis showed that viruses treated with 500 µg.mL^−1^ of *A*. *theiformis* extract established infection in 5% of cells compared to the plant-extract-free virus, which infected 60% of cells (Fig. 2c,d). Treatment of virus with *A*. *theiformis* extract was more potent at inhibiting infection of both viral strains than EGCG (Fig. 2c,d). Furthermore, to detect single-stranded viral RNA (ZIKV ssRNA) produced during viral replication, fluorescence *in situ* hybridisation (FISH)-based assays coupled with immunofluorescence and confocal microscopy were performed on Vero cells infected with ZIKV-MR766 or ZIKV-PF13 for 24 hours. The FISH probe produced no signal in non-infected control cells and a bright signal in the cytoplasm of cells infected with both viral strains (Fig. 2e), validating its specificity. Incubation of ZIKV with *A*. *theiformis* extract resulted in a 10-fold reduction of the number of cells positive for ZIKV ssRNA, compared to mock-treated cells, regardless of the viral strain (Fig. 2f,g). Together, these results showed that *A*. *theiformis* exerts a potent antiviral effect against an African and an Asian ZIKV strains.

### *A*. *theiformis* extract inhibits ZIKV binding to the cell surface

**Figure 3:**
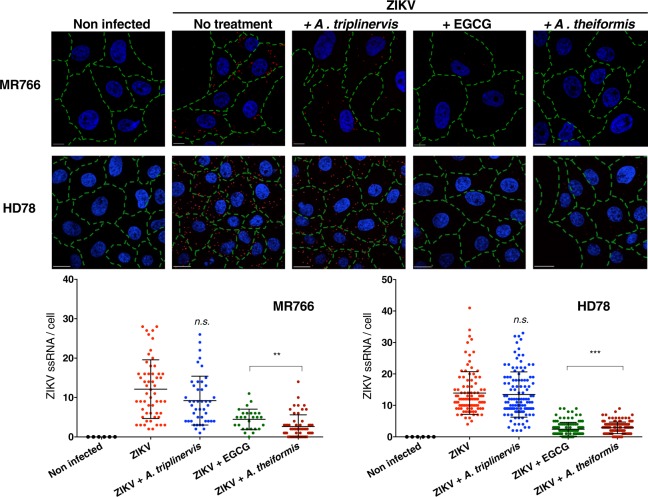
*A*. *theiformis* extract inhibits ZIKV entry to the cell surface. ZIKV-MR766 or ZIKV-HD78 were incubated with *A*. *theiformis* extract for 1 h at 37 °C. *A*. *triplinervis* and EGCG (100 µM) were used as negative and positive controls, respectively. Vero cells were incubated on ice with viruses at a MOI of 500. Cells were rinsed with cold PBS, shifted at 37 °C for 90 min and then fixed, permeabilised, processed for FISH using a probe specific for viral RNA (red) and finally stained with NucBlue to visualize nuclei (blue). Green lines outline the cell membranes stained with A488-conjugated wheat germ agglutinin. Scale bars are 20 μm. The images shown are representative of three independent experiments. Bottom panels: quantification of ZIKV ssRNA spots counted per cell (n ≥ 100).

Our data suggest that *A*. *theiformis* acts by interfering with at least one of the early steps of ZIKV infection. To determine which of these step(s) was/were affected, cell-binding assays were performed. These assays were performed with the MR766 strain and with HD78, another African ZIKV strain that replicated very efficiently in insect cells. MR766 and HD78 ZIKV particles were incubated 1 hour with *A*. *theiformis* extract and then added on cell monolayer at 4 °C to prevent viral internalisation. The cells were washed with ice-cold PBS to remove the unbound virus then shifted to 37 °C for 90 minutes. FISH assays coupled with confocal microscopy approaches were used to detect the presence of viral ssRNA in the cytoplasm of infected cells. Epigallocatechin gallate (EGCG) and *A*. *triplinervis* extracts were used as positive and negative controls, respectively. Around 15 molecules of viral RNA were detected in non-treated control cells infected with both viral strains (Fig. 3). *A*. *triplinervis* extract did not significantly affect viral entry since the number of ssRNA molecules per infected cell was similar than in control cells (Fig. 3). As expected from previous studies^24^, less ssRNA per cell was detected in the EGCG-treated preparations, as compared to control cells (Fig. 3). In the presence of *A*. *theiformis* extract, around 2 ssRNA molecules per infected cell were detected, which is around 6–7 times less than in control cells (Fig. 3). These results suggest that *A*. *theiformis* inhibits ZIKV penetration into Vero cells.

To further dissect which viral entry step was affected by *A*. *theiformis* extract, cell-attachment assays were performed with the African strain ZIKV-MR766^22^. Vero cells were incubated 1 hour at 4 °C with ZIKV particles at MOI of 1 in presence or in absence of *A*. *theiformis* extract and amounts of attached viruses were evaluated by RT-qPCR (Fig. S2). EGCG and *A*. *triplinervis* were used as positive and negative controls, respectively. A reduction of at least 95% of ZIKV attachment to the cell surface was observed with *A*. *theiformis* extract compared to untreated cells (Fig. S2).

**Figure 4:**
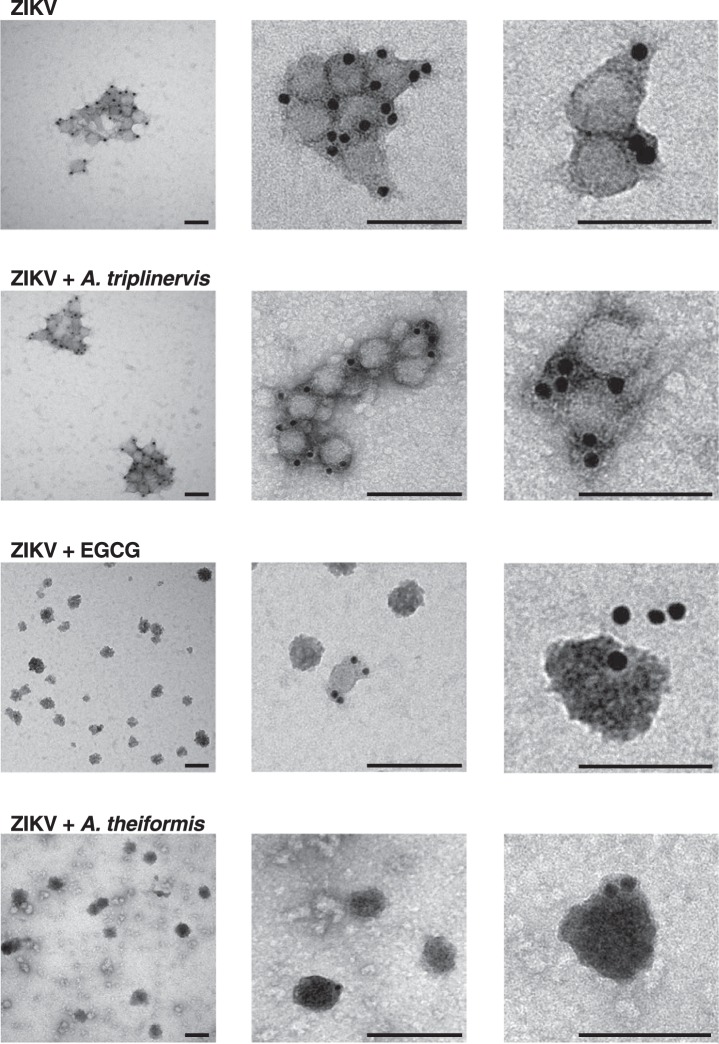
*A*. *theiformis* extract deforms ZIKV particles. ZIKV-HD78 was left untreated or was treated with *A*. *theiformis* extract for 15 min at 37 °C. *A*. *triplinervis* and EGCG (100 µM) were used as negative and positive controls, respectively. Samples were processed for immunogold labeling using anti-Env antibodies, negatively stained, and analysed by transmission electron microscopy. Scale bars are 50 nm. The images shown are representative of two independent experiments.

To investigate the physical effects of *A*. *theiformis* on ZIKV morphology, negative staining electron microscopic (EM) studies were performed with a highly concentrated preparation of the HD78 ZIKV strain. The virus was incubated with *A*. *theiformis*, EGCG, *A*. *triplinervis* or PBS at 37 °C for 1 hour prior to EM analysis coupled to immunogold labelling of the viral E (Fig. 4). The diameter of the E-positive particles detected in the non-treated control fractions was around 40 to 50 nm (Fig. 4), which is in good agreement with the diameter of ZIKV particles^25^. The mock-treated virions were spherical. As expected from our previous results showing that *A*. *triplinervis*, had no antiviral effect on ZIKV infection (Fig. 1c), virions treated with *A*. *triplinervis* had a similar shape and diameter than non-treated control virions (Fig. 4). Incubation of the virus with either EGCG or *A*. *theiformis* deformed the viral shape (Fig. 4). Interestingly, EGCG or *A*. *theiformis* virions were less aggregated than plant-extract free or *A*. *triplinervis*-treated ones (Fig. 4). Moreover, EGCG or *A*. *theiformis* virions appeared darker than control virions, likely due the enhanced permeation of the staining solution into the viral core. The darkening effect was more prominent in the presence of *A*. *theiformis* than EGCG. This suggests that the viral lipid membrane was highly damaged by *A*. *theiformis* treatment. Together, these results suggest that *A*. *theiformis* extracts interact with the viral membrane and that this interaction may deform the viral shape and thus affect viral binding to the cell membrane. These EM data are thus in good agreement with our RT-qPCR analysis (Fig. S2).

### *A*. *theiformis* exhibited antiviral effect against 4 DENV clinical isolates and an epidemic Brazilian ZIKV strain in human cells

**Figure 5:**
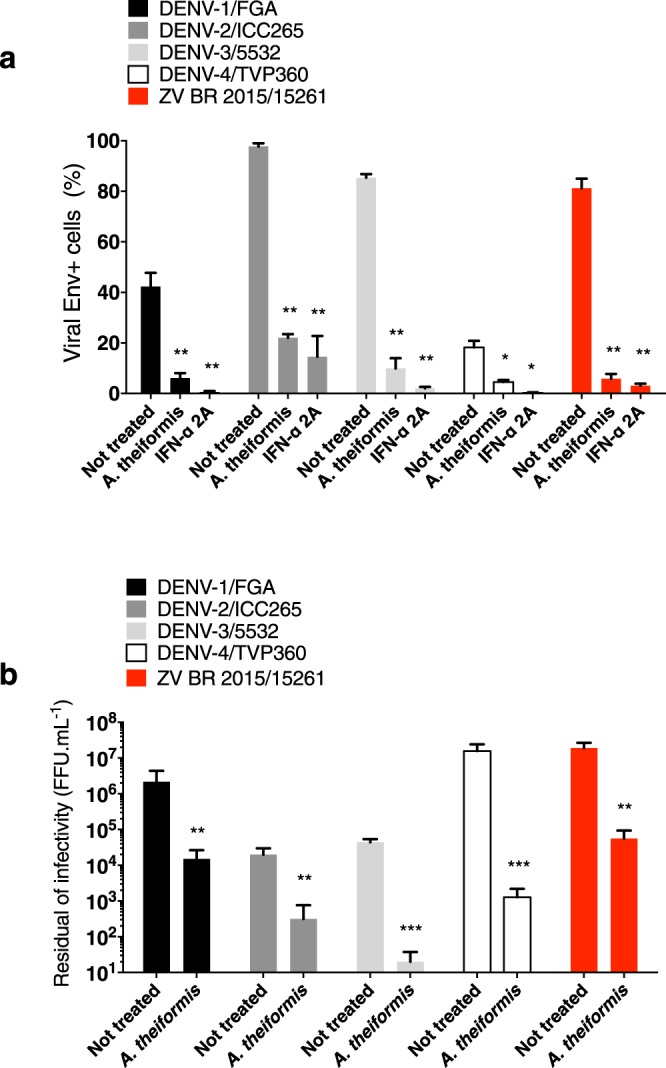
*A*. *theiformis* extract exerts antiviral effect against 4 DENV clinical isolates and an epidemic Brazilian ZIKV strain. Four DENV serotypes (DENV-1, 2, 3 and 4) and a Brazilian epidemic strain of ZIKV (ZV BR2015/15261) were incubated with *A*. *theiformis* extract for 1 h at 37 °C. (**a**) Huh7.5 cells were left uninfected or were infected with DENV at an MOI of 0.1 or with ZIKV at an MOI of 0.4 for 48 h. Recombinant IFN-α 2A (200 IU.mL^−1^) was added 2 h post infection and used as positive control. The percentage of E positive cells was evaluated using the Operetta High-Content Imaging System (PerkinElmer). Results are means ± SD of three independent experiments. (**b**) The residual infectivity of treated particles was titrated in C6/36 cells using a foci-forming immunodetection assay. Data represent the means ± SD from three independent experiments. One-way ANOVA and Dunnett’s test (*p < 0.05; **p < 0.01; ***p < 0.001).

We next wondered if *A*. *theiformis* exert antiviral activity against DENV another medically relevant flavivirus. The potential anti-DENV activity of *A*. *theiformis* was evaluated using clinical isolates representing the 4 different serotypes. Recombinant interferon (IFN)-α 2A, which are known to block DENV replication^26^ was used as positive control. To validate further the anti-ZIKV activities of *A*. *theiformis*, a clinical isolate of Zika virus (ZV BR 2015/15261) that was responsible for the 2016 epidemic in Brazil was also used in this experiment. *A*. *theiformis* (500 µg.mL^−1^) was pre-incubated with DENV or ZIKV for 1 hour at 37 °C then the mixture was used to infect Huh7.5 cells. After 72 h, immunofluorescence assays using antibodies against the E protein were performed to identify infected cells. The number of E positive cells was evaluated using the Operetta High-Content Imaging System (PerkinElmer)^27^. *A*. *theiformis* extract showed potent antiviral activity against the four DENV serotypes tested (Fig. 5a). DENV-1, 2 and 3 were the more sensitive to *A*. *theiformis* than DENV-4, with a 70 to 80% reduction of E-positive cells (Fig. 5a). *A*. *theiformis* also showed a robust antiviral effect against the ZIKV Brazilian epidemic strain (Fig. 5a).

To establish a causal relationship between the reduced percentages of E-positive cells in the samples treated with *A*. *theiformis* extract and a loss of DENV and ZIKV-BR infectivity, the presence of infectious viral particles in the inoculum was titrated by foci-forming immunodetection assay. DENV and ZIKV-BR virions treated with non-cytotoxic concentration of *A*. *theiformi*s extracts lost up to 3-log infectivity (Fig. 5b). Together, our results show that *A*. *theiformis* exhibits antiviral effect against 4 DENV clinical isolates and an epidemic Brazilian ZIKV strain in human cells.

## Discussion

In the absence of effective antivirals or vaccines against ZIKV, this arbovirus has emerged as a serious health burden that affected millions of people in the past 2–3 years. The development of antiviral agents from synthetic sources requires significant amounts of effort for drug design and validation. The use of plants as sources of antiviral drugs to treat viral infections represents instead a more economical, simple and environmental-friendly way^28,29^. Chemical substances are classified as virucidal agents if they are able to inactivate the extracellular virions, either by damaging the protein coat or penetrating the virion or by destroying the viral genome; hence, resulting in decreased infectivity of the virus^30^. We demonstrated here, using a panel of assays, that four strains of ZIKV exhibited decreased infectivity and thus viral replication ability when incubated with *A*. *theiformis* extract. Therefore, *A*. *theiformis* extract is a suitable candidate for the development of Zika fever treatment. *A*. *theiformis* was also active against four DENV clinical strains.

Our FISH and RT-qPCR data demonstrated that *A*. *theiformis* potently inhibited ZIKV attachment to the host cell. Moreover, EM observations clearly showed that *A*. *theiformis-*treated ZIKV-virions differed in shape from plant-extract-free particles. In the presence of *A*. *theiformis* extract, ZIKV were also less prone to aggregation than plant-extract-free ones. Finally, *A*. *theiformis* treatment seemed to render viral particles more permeable to the EM staining solution. These FISH and EM data showed that the active compounds present in *A*. *theiformis* acts on the virion itself, probably by binding viral E proteins and/or phosphatidylserine exposed at the surface of the virions. Since the anti-E antibodies 4G2 were able to bind *A*. *theiformis*-treated virions, the conformation of E was probably not affected by the treatment. We thus favour the hypothesis of a direct interaction between *A*. *theiformis* compounds and phosphatidylserine exposed at the surface of the virus. Such interactions might alter the conformation of phosphatidylserines, which in turn, could inhibit the docking of the virus to the host cell surface.

EGCG, which belongs to the flavonoid family, is a promising candidate for the development of a new class of broad-spectrum antiviral. It exerts its antiviral effects by inhibiting the entry into the host cell of a panel of unrelated RNA and DNA viruses, such as ZIKV, DENV, WNV, hepatitis C virus, influenza virus, herpes simplex virus (HSV) and hepatitis B virus^24,31–34^. EGCG induces physical damage to influenza virus and HSV particles, which may result in the loss of their attachment capacity to the host-cell membrane^33,34^. Using ligand-docking and molecular dynamic simulations, an *in silico* study has predicted the presence of an EGCG binding site within the ZIKV E protein^15^. We proposed that *A*. *theiformis extract* acts through a similar mechanism than EGCG, probably via binding to the surface of the virions. Our flow cytometry, FISH, RT-qPCR and titration experiments showed that *A*. *theiformis* extract was as potent as EGCG in inhibiting ZIKV infection.

*A*. *theiformis*, which is widely distributed across the Mascarene Islands, is currently the only known representative of the *Aphloiaceae* family^35^. It is consumed as herb tea and is therefore safe for humans. It is believed to have a large number of medicinal properties^16,36–38^, including anti-inflammatory activities. Our phytochemical analysis of *A*. *theiformis* extract using an ultra-high-performance liquid chromatography-diode array detector-tandem mass spectrometry UHPLC-DAD-MS^39^ (Fig. S3) showed its richness in phenolic compounds, mainly C-glycosylated xanthones, such as mangiferin and flavonoids (Table S1). Phenolic compounds from plant extracts possess many beneficial properties; including antiviral activities^24^. Polyphenols such as delphinidin, baicalein and naringin are known to inhibit the early steps of WNV, ZIKV and DENV replication^40–42^. Thus, it is likely that one or more of *A*. *theiformis* polyphenols are responsible for the anti-ZIKV and -DENV activities reported here. It would be of great interest to determine which components among the identified polyphenols are responsible for the antiviral activity of *A*. *theiformis* or whether there is a synergetic effect of several compounds leading to anti-ZIKV activity. Isolation, characterization and chromatography-based purification will be thus performed in order to identify the active components from the crude extract. It would also be interesting to compare the phytochemical composition of *A*. *theiformis* extract with those of *H*. *ambavilla* and *A*. *triplinervis* to develop structure–activity relationship (SAR) studies. Such SAR studies would broaden our understanding of family of compounds that contribute to the antiviral action against medically-important pathogens such as ZIKV and other related mosquito-borne viruses.

In summary, we showed that *A*. *theiformis* extract exerts antiviral effect against historical and contemporary strains of ZIKV and four DENV serotypes in mammalian cells by impairing the attachment of the virions to the host cell membrane. *A*. *theiformis*, an indigenous plant from Reunion island, may thus represent a potential prophylactic agent targeting the entry of two medically relevant flavivirus and could thus be used to treat patients.

## Materials and Methods

### Cell lines, virus strains and infection

Vero cells (ATCC, CCL-81) and human-derived Huh7.5 hepatoma cells (ATCC, PTA-8561TM; Manassas, Virginia, USA) were cultured in minimum essential medium (MEM: Gibco/Invitrogen, Carlsbad, CA) supplemented with 5% heat-inactivated foetal bovine serum (FBS), 2 mmol.L^−1^ L-Glutamine, 1 mmol.L^−1^ sodium pyruvate, 100 U.mL^−1^ of penicillin, 0.1 mg.mL^−1^ of streptomycin and 0.5 µg.mL^−1^ of fungizone (PAN Biotech) under a 5% CO_2_ atmosphere at 37 °C. C6/36 *Aedes albopictus* cells (ATCC CRL-1660, Manassas, Virginia, USA) were maintained at 27 °C in Leibovitz’s L-15 medium supplemented with 10% heat-inactivated FBS, 1% P/S, 1% MEM Non-Essential Amino Acids Solution (Gibco) and 2% Tryptose Phosphate Browth (Gibco).

The clinical isolate PF-25013-18 of ZIKV (ZIKV-PF13) has been previously described^23^ and was amplified on Vero cells. ZIKV-MR766 and GFP-expressing strain of ZIKV-MR766 (ZIKV_GFP_) are molecular clones of the historical strain MR766 of ZIKV^22^. Zika strain HD78788 was obtained from the Biological Resource Centre of Pasteur Institute and was grown on C6/36 cells. The clinical isolate of Zika virus (ZK BR 2015/15261) was isolated from a patient with Zika fever from Northeast of Brazil in 2015 and was amplified on C6/36 cells. DENV-1/FGA/89 was isolated in 1989 from a South American patient suffering from DF (GenBank: AF226687). DENV-2/ICC-265 was isolated from a DF patient in Brazil in 2009. DENV3/5532 was isolated in 2007 from a fatal case of dengue with visceral manifestations in a patient in Paraguay (GenBank: HG235027). DENV-4/TVP360 is a laboratory strain that was kindly provided by Dr. Ricardo Galler (Fundação Oswaldo Cruz, Rio de Janeiro, Brazil; GenBank: KU513442). DENV stocks were grown in C6/36 cells and titrated by foci-forming immunodetection assay.

Cells were infected with the different viruses at a multiplicity of infection of 1, unless stated otherwise.

### Antibody

Anti-*pan* flavivirus E monoclonal antibody 4G2 coupled with Alexa Fluor 594 was purchased from RD Biotech.

### Plant material

Fresh aerial parts of *Aphloia theiformis*, *Hubertia ambavilla* and *Ayapana triplinervis* were collected in Reunion Island in January 2016. The voucher specimens were deposited in the Herbarium of the University of Reunion Island.

### Extraction of plant material

Fresh aerial parts of *Aphloia theiformis*, *Hubertia ambavilla* and *Ayapana triplinervis* were submitted to solvent-free microwave extraction. The extracts were recovered by gravity after heating 500 g of plant material at 800 W for 20 min at atmospheric pressure using IDCO E200 extractor (IDCO SAS, Marseille, France). After filtration, aqueous phase was lyophilised using cryotec 20 K (Cryotec, Saint-Gély-du-Fesc, France) to produce a brown powder. The crude extracts were solubilised in sterile phosphate buffer saline (PBS) and stored at −80 °C until used for the antiviral assays.

### MTT assay

Vero or Huh7.5 cells were cultured in 96-well culture plate at a density of 1 × 10^4^ cells per well and treated with two fold dilutions of plant extracts ranging from 2000 µg.mL^−1^ to 30 µg.mL^−1^. After an incubation period of 72 h at 37 °C, cells were rinsed with PBS 1× and 120 µL of culture medium mixed with 5 mg.mL^−1^ MTT (3-[4,5-dimethylthiazol-2-yl]-2,5- diphenyltetrazolium bromide) solution was added. Incubation was extended for 2 h, then MTT medium was removed and the formazan crystals were solubilised with 100 µL of Dimethyl sulfoxide (DMSO). Absorbance was measured at 570 nm with a background subtraction at 690 nm.

### Flow cytometry assay

Cells were fixed with 3.7% paraformaldehyde (PFA) for 20 min, washed twice with PBS and then submitted to a flow cytometric analysis using FACScan flow cytometer (Becton Dickinson). Results were analysed using the Flowjo software.

### Cell cycle analysis

Vero cells were seeded at 2 × 10^5^ cells/well in glass bottom 6-well plates. Cell cycle distribution was determined by flow cytometry^43^. Cells were trypsinised and fixed in 70% ethanol at −20 °C for at least 2 h. Cells were re-suspended in PBS containing 40 µg.mL^−1^ propidium iodide and 100 µg.mL^−1^ RNase A. After incubation for 1 h at 37 °C cells were characterised. Data were acquired using the CytExpert software for CytoFLEX (Beckman Coulter). For each experiment, 10^4^ cells were analysed.

### Immunofluorescence assay

Cells grown on coverslips were fixed with 3.7% of PFA at room temperature (RT) for 10 min. Fixed cells were permeabilised with Triton X-100 (0.15%) in PBS for 4 min and stained using the mouse anti-pan flavivirus envelope E protein mAb 4G2 (1:1,000 dilution). Nucleus was stained with DAPI. The coverslips were mounted with Vectashield (Vector Labs), and fluorescence was observed using a Nikon Eclipse E2000-U microscope. Images were captured and processed using a Hamamatsu ORCA-ER camera and the imaging software NIS-Element AR (Nikon).

### Plaque-forming assay

Virus infectious titre was quantified using plaque forming unit assay. Vero cells were seeded in 48-well culture plates at a density of 3 × 10^4^ cells per well and incubated overnight at 37 °C to produce confluent monolayers. Ten-fold serial dilutions of supernatant were prepared in duplicate in culture medium and 0.1 mL of each dilution was added to the cells. Plates were incubated for 2 h at 37 °C, then 0.2 mL of culture medium supplemented with 5% foetal bovine serum (FBS) and 0.8% carboxymethylcellulose sodium salt (Sigma-Aldrich, France) were added, followed by a 4 days incubation at 37 °C. Then, media were removed and cells were fixed (PFA, 3.7%) and stained with 0.5% crystal violet (Sigma-Aldrich, France) diluted in 20% ethanol. Plaques were counted and expressed as plaque-forming unit per mL (PFU.mL^−1^).

### Foci-forming immunodetection assay

Foci-forming immunodetection assay in C6/36 cells was performed by seeding 1 × 10^5^ cells in 24 well plates and incubated overnight at 37 °C. Ten-fold serial dilutions of supernatant were prepared in duplicate in culture medium and 0.4 mL of each dilution was added to cells. After 1 h 30 min, the inoculum was removed and a CMC overlay media (L-15 supplemented with 10% FBS, 0.52% tryptose, 50 mg.mL^−1^ gentamicin, 1.6% carboxymethylcellulose) was added. The immunostaining was performed after seven days using the mouse monoclonal antibody 4G2 followed by goat-antimouse immunoglobulin conjugated to alkaline phosphatase (Promega, Madison, WI, USA), which was detected by adding a solution of NBT (nitroblue tetrazolium chloride) and BCIP (5- bromo-4-chloro-39-indolyphosphate p-toluidine salt) (Promega, Madison, WI, USA) as a substrate. Foci were counted and expressed as FFU.mL^−1^.

### Virus inactivation assay

Plant extracts were mixed with ZIKV_GFP_ (5 × 10^6^ PFU.mL^−1^) and then incubated at 37 °C for 1 h. The mixture was then diluted 50-fold (final virus concentration, 10^5^ PFU/well) with MEM, and the virus innocula subsequently added to monolayer of Vero cells seeded in 6-well plates. As control, ZIKV_GFP_ was mixed with plant extracts, immediately to 50-fold diluted (no incubation period), and added to Vero cells for infection. After 2 h of adsorption at 37 °C, the diluted inocula were discarded, and the cells were washed twice with PBS. The cells were further incubated with fresh medium at 37 °C for 24 h before being subjected to flow cytometry as above described.

### Negative and immunogold staining of ZIKV particles

Clarified supernatants of HD78-infeted C6/36 cells were incubated for 10 min with plant extracts at RT (*A*. *triplinervis* and *A*. *theiformis* at a final concentration of 500 μg.mL^−1^, EGCG at a final concentration of 50 µM). The preparations were filtered through a 0.45 µM filter, absorbed into a formvar/carbon coated negatively discharged EM grids for 10 min at RT and then fixed for 20 min in PFA 4%. Samples were quenched with 50 mM NH_4_Cl, blocked in PBS with 1% BSA and immunolabeled with anti-Env MAb for 20 min in PBS with 1% BSA followed by protein A-gold (10 nm) treatment for 15 min. Finally, samples were fixed with 2.5% Glutaraldehyde in 1X Sodium Cacodylate buffer, stained for 1 min with 2% aqueous uranyl acetate, washed for three times with ultrapure water and dried for 30 min. Images were recorded with TECNAI SPIRIT 120Kv equipped with a bottom-mounted EAGLE 4Kx4K camera.

### Quantification of the number of infected cells by fluorescence *In Situ* Hybridization (FISH) and confocal analysis

Vero cells were seeded at 0.5 × 10^4^ cells/well in glass bottom 96-well plates (Eppendorf) and infected with ZKV-PF13 or MR766 strains at MOI of 1 for 24 h. Alternatively, ZIKV strains were pre-incubated for 1 h at 37 °C with plant extracts in DMEM before infection (*A*. *triplinervis* and *A*. *theiformis* at a final concentration of 500 μg.mL^−1^, EGCG at a final concentration of 50 µM). After fixation with 4% PFA for 30 min at RT, cells were stained with wheat germ agglutinin (WGA) 488 conjugate (Thermo Fisher) in PBS-BSA 0.5% over night at 4 °C. After immunostaining, ZIKV plus strand RNA from both strains was detected following the manufacturer’s protocol (ViewRNA ISH Cells Assays) using probe sets designed by Affymetrix. Alexa-Fluor 546-conjugated ZIKV probe set recognizes a region between nt position 2 and 1144 of the ZIKV genome. Nuclei were stained with NucBlue (Thermo Fisher Scientific) for 15 min at RT. Images were acquired with a Zeiss LSM 700 laser scanning confocal microscope equipped with a X63 objective. Images were processed with Zen2 (blue edition) software and analysed with the ICY software (icy.bioimageanalysis.org) using the Spot Detector plugin.

### Virus binding assay

Virus binding assay was performed as previously described^22^. Briefly, Vero cells were seeded at 2 × 10^5^ cells/well in 6-well plates. Cell monolayers were washed in cold PBS and cooled at 4 °C at least 30 min in presence of cold MEM supplemented with 5% FBS. Pre-chilled cells were incubated at 4 °C with MR766-ZIKV at MOI of 1 with or without of *A*. *theiformis* extract. After 1 h of incubation, the virus input was removed and the cell monolayers were washed with cold MEM supplemented with 2% FBS. Samples were then subjected to RT-qPCR.

### RT-qPCR

Total RNA including genomic viral RNA was extracted from cells with RNeasy kit (Qiagen) and reverse transcribed using E reverse primer and M-MLV reverse transcriptase (Life Technologies) at 42 °C for 50 min. Quantitative PCR was performed on a CFX96 Real-Time PCR Detection System (Bio-Rad). Briefly, cDNA were amplified using 0.2 μM of each primer and 1× GoTaq Master Mix (Promega). For each single-well amplification reaction, a threshold cycle (Ct) was calculated using the CFX96 program (Bio-Rad) in the exponential phase of amplification. Relative changes in gene expression were determined using the ΔΔ*Ct* method and reported relative to the control. The primers used in this study were previously reported in^22^. A synthetic gene coding for nucleotides 954 to 1306 of the MR766 strain (GenBank: LC002520) cloned in the pUC57 plasmid was used as template to generate a standard curve, which then served to make absolute quantitation of bound viruses.

### Virus entry assay

Vero cells were seeded at 1.2 × 10^4^ cells/well in glass bottom 96-well plates (Eppendorf) and infected with ZIKV HD78 or MR766 strains at MOI of 500 for 1 h at 4 °C. Alternatively, ZIKV strains were pre-incubated for 1 h at 37 °C with plant extracts in DMEM before infection (*A*. *triplinervis* and *A*. *theiformis* at a final concentration of 500 μg.mL^−1^, EGCG at a final concentration of 50 µM). Cells were then shifted at 37 °C for 90 min and fixed with 4% PFA for 30 min at RT. FISH assays were performed as indicated above.

### Statistical analysis

Comparison between different concentrations was done by a one-way ANOVA test. All values were expressed as mean ± SD of at least three independent experiments. All statistical tests were done using the software Graph-Pad Prism version 6.0. Values of p < 0.05 were considered statistically significant for a Dunnett’s multiple comparisons test. Degrees of significance are indicated on the figure as follow: *p < 0.05; **p < 0.01; ***p < 0.001, ****p < 0.0001, ns = not significant.

## Electronic supplementary material

Supplementary information
