# Supplementary material for: Extract from Aphloia theiformis, an edible indigenous plant from Reunion Island, impairs Zika virus attachment to the host cell surface
Source: Sci Rep. 2018 Jul 18;8:10856. doi: 10.1038/s41598-018-29183-2 (PMC6052117; doi:10.1038/s41598-018-29183-2)
Supplement: Supplementary file 1 — Supplementary information [file 41598_2018_29183_MOESM1_ESM.pdf]

Supplementary Information:

Extract from *Aphloia theiformis*, an edible indigenous plant from Reunion Island, impairs Zika virus attachment to the host cell surface

Elodie Clain[1#], Laura Sinigaglia[2#], Andrea Cristine Koishi[3], Olivier Gorgette[4], Gilles Gadea[1], Wildriss Viranaicken[1], Pascale Krejbich-Trotot[1], Patrick Mavingui[1], Philippe Desprès[1], Claudia Nunes Duarte dos Santos[3], Pascale Guiraud[1], Nolwenn Jouvenet[2] and Chaker El Kalamouni[1*]

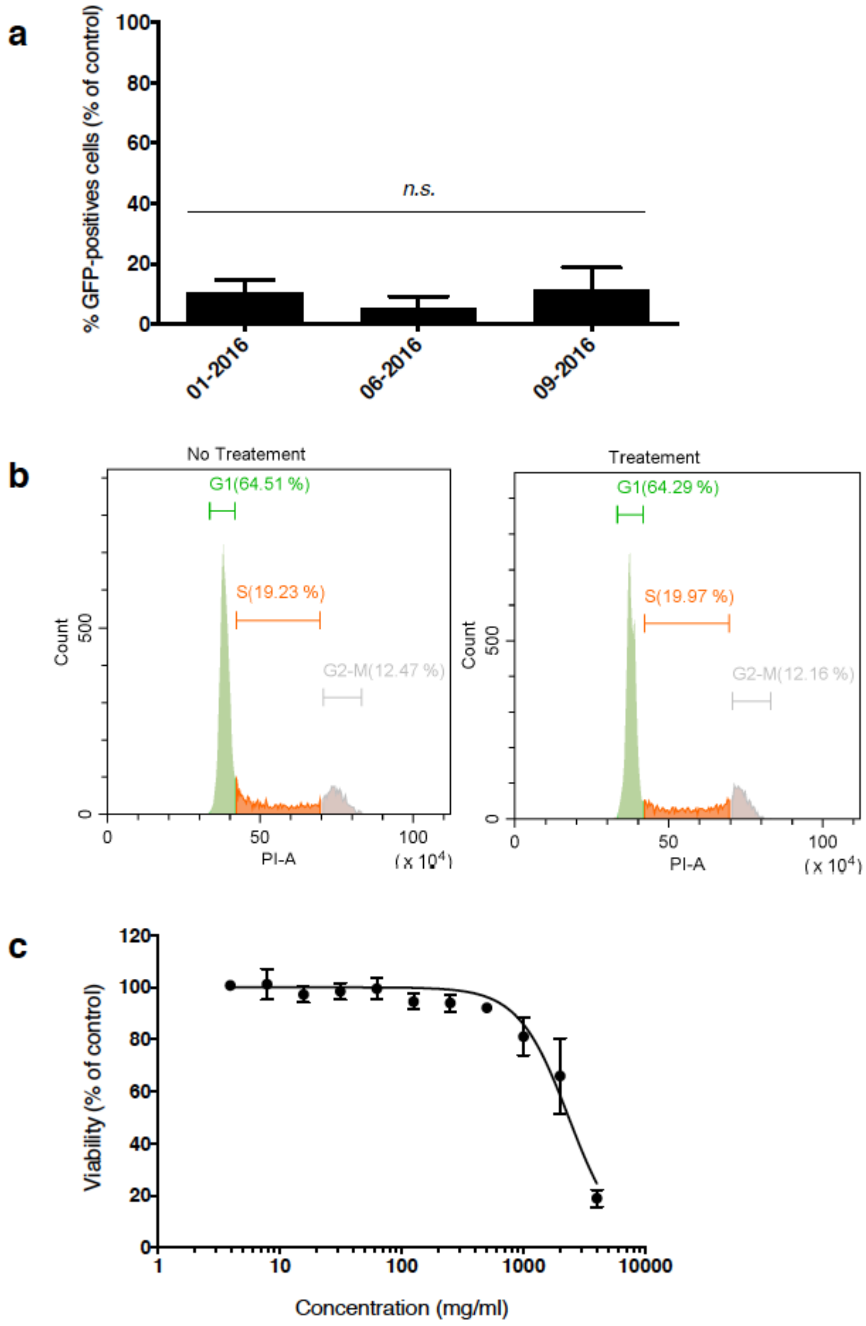

Figure S1. *A. theiformis* extract do not perturb cell growth. **(a)** Antiviral activity of *A. theiformis* extract did not vary significantly from one extraction to the other. GFP expression in Vero cells infected with ZIKV$_{GFP}$ (MOI 1) and treated with *A. theiformis* extract (500 µg.mL^{-1}) obtained from three different harvests in the year 2016 (01-2016, 06-2016 and 09-2016). Flow cytometric analysis of GFP fluorescence was performed 24 hpi. Results are means ± SD of three independent experiments and are expressed as relative value compared to untreated infected cells. **(b)** Vero cells were left untreated or were treated with 500 µg.mL^{-1} of *A. theiformis* extract for 24 h. Following cell staining with propidium iodide, cell cycle distribution was analysed by flow cytometry assay. Data was acquired using a CytExpert software for CytoFLEX. For each experiment, 10^4 cells were analysed. **(c)** Viability of Huh7.5 cells incubated with different concentrations of *A. theiformis* extract for 72 h. Cell metabolic activity was evaluated by MTT assay. Results are means ± SD of three independent experiments and are expressed as relative value compared to untreated cells.

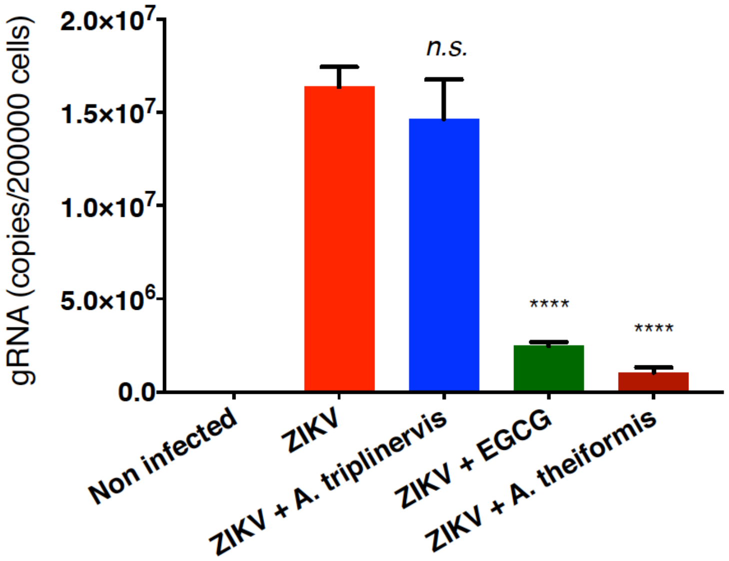

Figure S2. *A. theiformis* extract inhibits ZIKV attachment to the cell surface. Vero cells were infected with ZIKV-MR766 at MOI of 1 for 1 h at 4°C with or without 500 µg.mL^{-1} of *A. theiformis* extract. *A. triplinervis* and EGCG (100 µM) were used as negative and positive controls, respectively. The number of virus particles bound to cell surface was measured by RT-qPCR. Values represent the mean and standard error from triplicate.

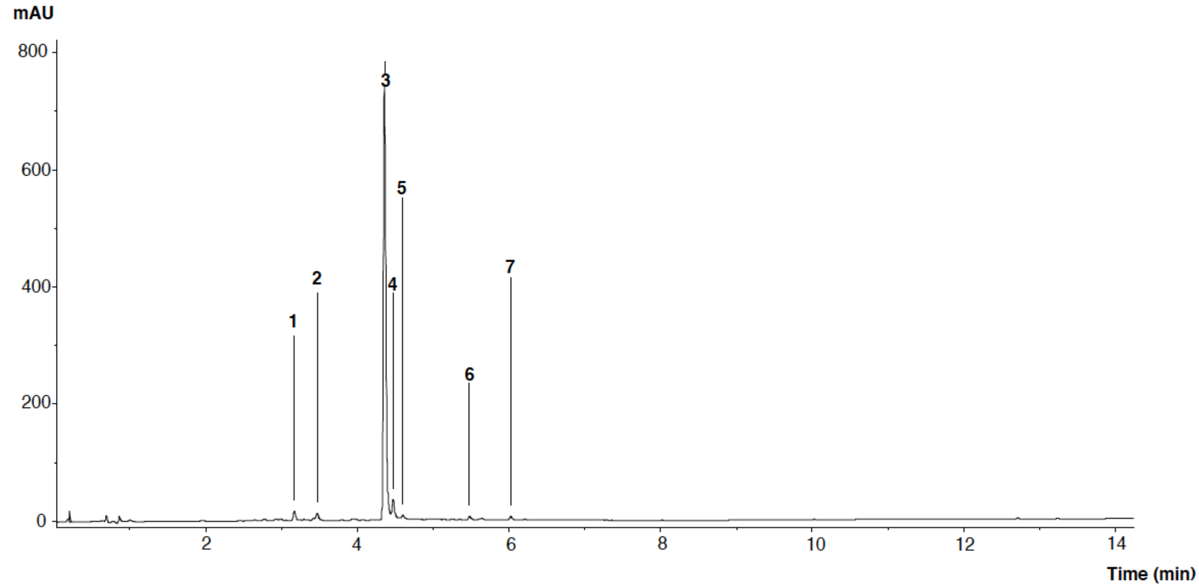

Figure S3. UHPLC-DAD chromatogram of *Aphloia theiformis* extract at 260 nm. *A. theiformis* extract was characterized by UHPLC apparatus (Agilent 1290, Santa Clara, CA) equipped with an Agilent Zorbax SB-C18 (100 nm x 2.1 nm x 1.8 µm) column and an UV-VIS-DAD. The UHPLC apparatus was coupled with an Esquire 6000 ion trap mass spectrometer using an ESI source (Bruker Daltonics, Billerica, MA). Alternating negative and positive mode was performed for analysis. *A. theiformis* extract was dissolved in MeOH-H_2O mixture (1/1, v/v) at 1 mg.mL^{-1}. Analyses were conducted in triplicate.

Peaks	Compounds	t_R (min)	$[M-H]^-$ (m/z)	MS/MS fragments	Concentration w/w (in %)
1	Neomangiferin	3.2	583	565, 493, 463, 421, 331	0.15
2	Iriflophenon-C-glucoside*	3.5	407	317, 287	nq
3	Mangiferin	4.4	421	403, 331, 301	10.00
4	ni**	4.5	437	419, 347, 317	0.47
5	Isomangiferin	4.6	421	403, 331, 301	0.07
6	Homomangiferin	5.5	435	417, 345, 315, 272	0.02
7	Aspalathin*	6.0	451	433, 361, 331, 287	nq

Table S1: Identification of polyphenols from *A. theiformis* extract
ni : not identified; nq: not quantified
* Tentatively identified
** Seems to be an Homomangiferin derivative
